# Characterization of circular RNAs in dorsal root ganglia after central and peripheral axon injuries

**DOI:** 10.3389/fncel.2022.1046050

**Published:** 2022-12-12

**Authors:** Hong-Jun Cao, Li Huang, Meng-Ru Zheng, Tao Zhang, Ling-Chi Xu

**Affiliations:** ^1^Jiangsu Clinical Medicine Center of Tissue Engineering and Nerve Injury Repair, Co-Innovation Center of Neuroregeneration, Nantong University, Nantong, Jiangsu, China; ^2^School of Medicine and Holistic Integrative Medicine, Nanjing University of Chinese Medicine, Nanjing, Jiangsu, China

**Keywords:** circular RNAs, dorsal root ganglion, nerve regeneration, central axon injury, peripheral axon injury

## Abstract

In central nervous system, axons fail to regenerate after injury while in peripheral nervous system, axons retain certain regenerative ability. Dorsal root ganglion (DRG) neuron has an ascending central axon branch and a descending peripheral axon branch stemming from one single axon and serves as a suitable model for the comparison of growth competence following central and peripheral axon injuries. Molecular alterations underpin different injury responses of DRG branches have been investigated from many aspects, such as coding gene expression, chromatin accessibility, and histone acetylation. However, changes of circular RNAs are poorly characterized. In the present study, we comprehensively investigate circular RNA expressions in DRGs after rat central and peripheral axon injuries using sequencing analysis and identify a total of 33 differentially expressed circular RNAs after central branch injury as well as 55 differentially expressed circular RNAs after peripheral branch injury. Functional enrichment of host genes of differentially expressed circular RNAs demonstrate the participation of Hippo signaling pathway and Notch signaling pathway after both central and peripheral axon injuries. Circular RNA changes after central axon injury are also linked with apoptosis and cellular junction while changes after peripheral axon injury are associated with metabolism and PTEN-related pathways. Altogether, the present study offers a systematic evaluation of alterations of circular RNAs in rat DRGs following injuries to the central and peripheral axon branches and contributes to the deciphering of essential biological activities and mechanisms behind successful nerve regeneration.

## Introduction

Axon injury elicits distal nerve degeneration, impairs neuronal functions, and causes motor incapacity, sensation loss, and neuropathic pain (Stoll and Muller, 1999; Hu, 2016). The recovery effects and consequences of axon injury largely rely on the special localizations of injured axons. In the mammalian central nervous system, injured axons fail to regenerate toward their original targets and thus permanent functional disability is commonly observed after central nerve injury (Mahar and Cavalli, 2018). On the contrast, in the peripheral nervous system, injured axons preserve certain regenerative abilities and functional recovery of injured peripheral nerves may be achieved, especially after mild peripheral nerve injury, such as nerve crush and nerve injury with short gaps (He and Jin, 2016).

Dorsal root ganglion (DRG) neurons are pseudounipolar neurons that have two separate axonal branches stemming from one single axon. The central branch extends into the central nervous system and is generally incapable of regeneration after axon injury while the peripheral branch projects to peripheral target and often obtains a remarkable growth capacity (Neumann and Woolf, 1999; Nascimento et al., 2018). Despite different local microenvironment of central and peripheral axon, such as surrounding oligodendrocytes, astrocytes, and microglial cells in central nerves versus Schwann cells in peripheral nerves, the intrinsic regenerative status of DRG neurons are distinct after injury to central and peripheral branches (Hoffman, 2010; Renthal et al., 2020). Injury to the two separate axon branches generates a useful model for the direct comparisons of molecular alterations in DRGs and offers valuable insights into failed central axon growth and successful peripheral axon growth.

Recently, the application of high-throughput RNA sequencing discriminates genetic changes in DRGs after central and peripheral axon injury and reveals the essential roles of reactive oxygen species in axon regeneration by comparing changes between non-regenerative central axon injury and regenerative peripheral axon injury (Hervera et al., 2018). ATAC and ChIP sequencing explore underlying epigenomic characteristics (Palmisano et al., 2019). Recent data further decipher distinct cellular changes in DRGs after central axon injury and peripheral axon injury using single cell sequencing (Avraham et al., 2021). However, the unique features of circular RNAs in DRGs following central and peripheral axon injuries are less understood.

Circular RNAs are endogenous non-coding RNAs with covalently closed loop structures formed by transcript back splicing (Zhang et al., 2014; Chen and Yang, 2015). Although the abundances of a large number of circular RNAs are lower than their counterpart linear RNAs, circular RNAs are generally stably expressed (Memczak et al., 2013; Zhang et al., 2019). Moreover, many circular RNAs exhibit distinctive tissue and development expression patterns and play important regulatory roles under various physiological and pathological conditions (Memczak et al., 2013). Here, we made a injury to DRG central axon branch as well as a injury to the peripheral axon branch, collected DRGs at 24 h after nerve injury, and investigated circular RNA signatures in response to central and peripheral axon injuries, aiming to decipher key elements for effective nerve regeneration from the aspect of regulatory RNAs.

## Materials and methods

### Animals

A total of 56 8-week-old male Sprague Dawley rats (∼200 g) were obtained from the Animal Center of Nantong University. Rats were randomly divided into central axon branch injury group, central axon branch sham surgery group, peripheral axon branch injury group, and peripheral axon branch sham surgery group, with 14 rats in each group.

Animal work was carried out in accordance with the guidelines of Nantong University Institutional Animal Care and Ethical approved by the Administration Committee of Experimental Animals, Nantong University, Jiangsu Province, China.

### Surgery procedures

Central and peripheral axon injuries were performed according to a previous publication with modifications (Avraham et al., 2021). Briefly, for central axon branch injury, after anesthesia, a small midline skin incision was made at the L2-L3 vertebral level, dura mater was removed, and dorsal root was cut. For central axon branch sham surgery, dura mater was removed while dorsal root was not injured. Rats subjected to central axon branch injury or central axon branch sham surgery were designated as DR-Exp and DR-Sham, respectively. For peripheral axon branch injury, after anesthesia, a skin incision was made on the lateral aspect of the mid-thigh of rat hind limb and sciatic nerve was cut. For peripheral axon branch sham surgery, sciatic nerve was exposed but not injured. Rats subjected to peripheral axon branch injury or peripheral axon branch sham surgery were designated as SN-Exp and SN-Sham, respectively. Rat L4-L5 DRGs were collected at 24 h after surgery and stored at −80°C.

### Sequencing analysis

Total RNAs was extracted from rat L4-L5 DRGs using TRIzol reagent kit (Invitrogen, Carlsbad, CA, USA) and subjected to RNA quality check using an Agilent Bioanalyzer (Agilent Technologies, Palo Alto, CA, USA). mRNA was enriched, fragmented into short fragments, and reverse transcripted to cDNA. cDNA fragments were ligated to Illumina sequencing adapters and sequencing was performed using HiSeq™ 4000 platform. RNA libraries were sequenced on the Illumina sequencing platform by Genedenovo Biotechnology Co., Ltd. (Guangzhou, China). Sequencing data were stored in Genome Sequence Archive database with accession number CRA006070.

For bioinformatic analysis, raw reads obtained from sequencing were filtered by fastp (version 0.18.0) to obtain high quality clean reads (Chen et al., 2018). Clean reads were mapped to the reference genome. Circular RNAs were identified using bioinformatic tools bowtie 2 and find_circ (Memczak et al., 2013). Identified circular RNAs were then filtered to obtain highly reliable data under following conditions: breakpoint equal to 1, anchor_overlap less than or equal to 2, edit less than or equal to 2, n_uniq greater than 2, best_qual_A greater than 35, or best_qual_B greater than 35, n_uniq greater than int (samples/2), and length less than 100 k. The abundances of circular RNAs were quantified using back-spliced reads per million mapped reads (RPM) according to the formula RPM = 10^6C/N, where C represented the back-spliced reads of target genes and N represented total back-spliced reads. RNA differential expression analysis was performed using edgeR by normalization and *p*-value calculation. Circular RNAs with log_2_ (fold change) > 1 or < −1 and *p*-value < 0.05 were screened and considered as differentially expressed. The functions of host genes of differentially expressed circular RNAs were discovered using kyoto encyclopedia of genes and genomes (KEGG) pathway enrichment analysis (Kanehisa and Goto, 2000), reactome enrichment analysis, and gene ontology (GO) term enrichment analysis (Croft et al., 2011; Fabregat et al., 2018). The significances of KEGG pathways, reactome pathways, or GO terms were calculated based on the numbers of all genes and candidate genes that with KEGG, reactome, or GO annotation as well as the numbers of all genes and candidate genes annotated to specific KEGG pathways, reactome pathways, or GO terms.

The competing endogenous RNA (ceRNA) network was constructed by assembling all co-expression competing circular RNAs, microRNAs, and mRNAs. Expression correlations between circular RNAs and microRNAs as well as microRNAs and mRNAs were analyzed using the Spearman Rank correlation coefficient (SCC) to screen negatively co-expressed circular RNA-microRNA pairs or microRNA-mRNA pairs. Expression correlations between circular RNAs and mRNAs were analyzed using Pearson correlation coefficient (PCC) to screen positively co-expressed circular RNA-mRNA pairs. The interactions between circular RNAs, microRNAs, and mRNAs in the ceRNA network were displayed using Cytoscape software (v3.6.0).

### Real-time PCR

Total RNA extracted from rat L4-L5 DRG was reversely transcribed to cDNA using PrimeScript RT Reagent Kit (TaKaRa Biotechnology Co., Ltd., Dalian, China). The characterization of circular RNA was determined using the divergent primers annealing at the distal end. PCR product was subjected to agarose gel electrophoresis and Sanger sequencing (Genewiz, Inc., Suzhou, China). The relative abundance of novel_circ_000290, novel_circ_001817 and novel_circ_000991 was determined using the comparative 2^–ΔΔCt^ method with GAPDH as the reference gene. Primer sequences were as follows (5′−3′): novel_circ_000290-F TGACTCCCTCTCTGGTGACA, novel_circ_000290-R GCTTCCTCAACACCATCACC, novel_circ_001817-F AGGCTATTCGCTTAGGATTCCA, novel_circ_001817-R CCAGGTAGTTGTGTCCGATGTA, novel_circ_000991-F CATCCACGTCATGAGGAACAG, novel_circ_000991-R TACAAGACACTCGGTCTACCAA, GAPDH-F ACAGCAACAGGGTGGTGGAC, and GAPDH-R TTTGAGGGTGCAGCGAACTT.

### Statistical analysis

Numerical data were expressed as mean with SEM. Comparisons between DR-Exp and DR-sham as well as SN-Exp and SN-sham were generated using unpaired *t*-test (GraphPad Prism 6.0 software, GraphPad Software, La Jolla, CA, USA). Significantly difference was set at a *p*-value < 0.05.

## Results

### Overview of circular RNA profiles in rat DRGs following central and peripheral axon injuries

**FIGURE 1:**
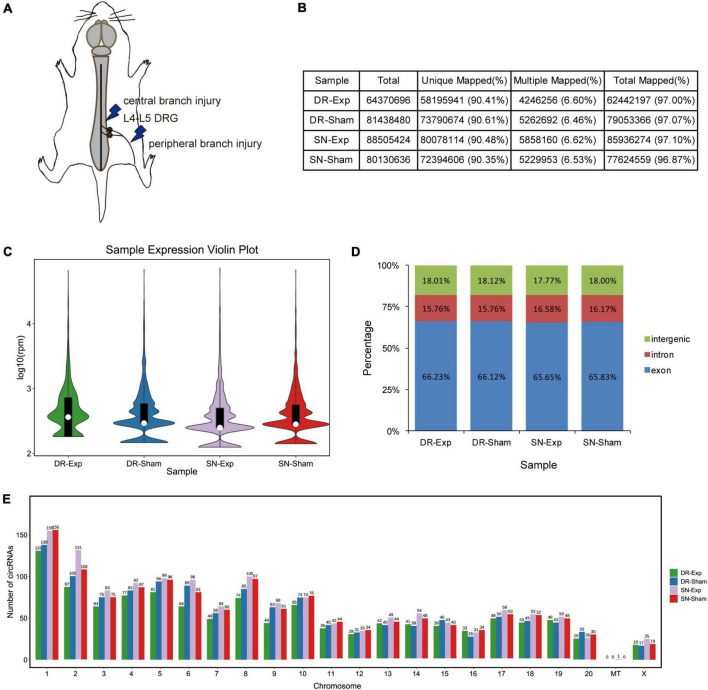
Characterization of circular RNAs in dorsal root ganglions (DRGs). **(A)** Schematic representation of nerve injury model. **(B)** The alignment of sequencing reads to the reference genome in rats underwent central injury, peripheral injury, or corresponding sham surgery. **(C)** The violin plot of the abundances of circular RNAs. **(D)** Genomic origins of circular RNAs. **(E)** Numbers of circular RNAs in each chromosome.

Dorsal root ganglions of rats underwent central axon branch injury, peripheral axon branch injury, or corresponding sham surgery were collected at 24 h after surgery and subjected to sequencing to obtain a global view of circular RNAs in rat DRGs after central and peripheral axon injuries (Figure 1A). Sequencing clean reads were mapped to approximately 97% of total reads. More than 90% mapped reads were uniquely mapped, indicating high read quality for RNA sequencing (Figure 1B).

Bioinformatic analysis identified a total of 2,310 circular RNAs in rat DRGs. These circular RNAs showed similar sample expression distributions after central and peripheral axon injuries (Figure 1C). The localizations of circular RNAs were also comparable in difference groups. About 18% of circular RNAs were derived from intergenic regions, about 16% were derived from intronic regions, while the majority were derived from exonic regions (Figure 1D). Most circular RNAs were localized in nuclear genome instead of the mitochondrial genome. Among all chromosomes, chromosome 1 contained the largest numbers of circular RNAs (Figure 1E).

### Identification of differentially expressed circular RNAs following central and peripheral axon injuries

**FIGURE 2:**
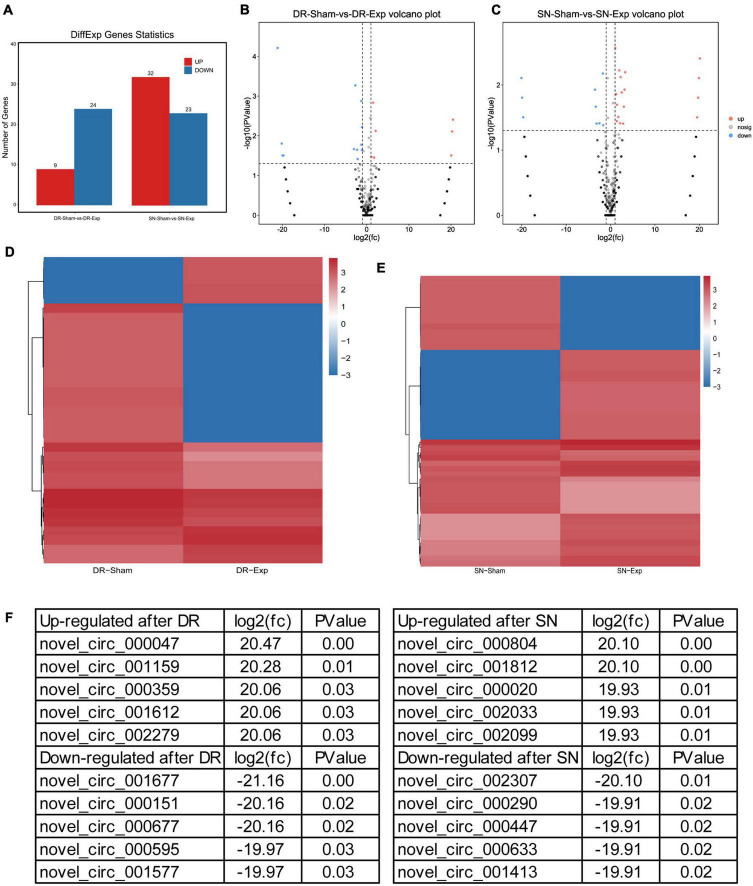
Changes of circular RNAs after rat central and peripheral axon injuries. **(A)** Numbers of differentially expressed circular RNAs after rat central and peripheral axon injuries. Red indicates up-regulated after nerve injury while blue indicates down-regulated after nerve injury. **(B,C)** The volcano plots of differentially expressed circular RNAs in DRGs after **(B)** central axon injury and **(C)** peripheral axon injury. **(D,E)** Heatmaps of differentially expressed circular RNAs in dorsal root ganglions (DRGs) after **(D)** central axon injury and **(E)** peripheral axon injury. Red indicates relatively high abundance and blue indicates relatively low abundance. **(F)** Top 5 up-regulated and top 5 down-regulated circular RNAs after rat central and peripheral axon injuries.

**FIGURE 3:**
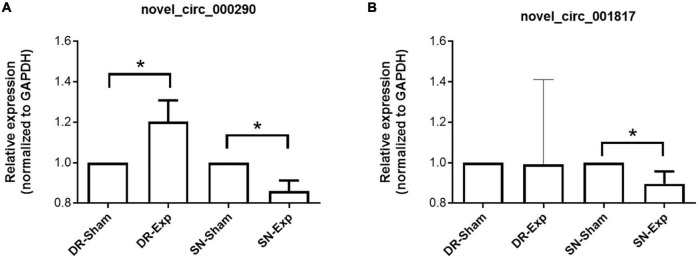
Validation of the expression of differentially expressed circular RNAs after rat central and peripheral axon injuries. Relative expression levels of novel_circ_000290 **(A)** and novel_circ_001817 **(B)** after rat central and peripheral axon injuries. Data are summarized from 3 experiments and presented as mean with SEM. *P-value < 0.05.

The expression levels of circular RNAs after central and peripheral axon injuries were determined to screen differentially expressed circular RNAs. A total of 33 circular RNAs were found to be differentially expressed after central branch injury versus central branch sham surgery, with 9 circular RNAs up-regulated and 24 circular RNAs down-regulated (Figures 2A, B). A larger number of circular RNAs showed different expression levels after peripheral branch injury versus peripheral branch sham surgery, with 32 circular RNAs up-regulated and 23 circular RNAs down-regulated (Figures 2A, C). The expression patterns of these differentially expressed circular RNAs were displayed in heatmaps (Figures 2D, E) and circular RNAs with the most robust changes were presented (Figure 2F). The abundances of these up-regulated or down-regulated circular RNAs were further examined using real-time PCR to validate the accuracy of sequencing analysis. PCR results showed that, in keeping with sequencing data, peripheral axon injuries reduced novel_circ_000290 and novel_circ_001817 expression, and central axon injuries induced novel_circ_000290 expression (Figure 3).

Detailed information of all differentially expressed circular RNAs, including their chromosome localizations, genomic start/end sites, spliced lengths, annotation types (intergenic, intronic, and exonic), and host genes, was provided in Supplementary Tables 1, 2.

**FIGURE 4:**
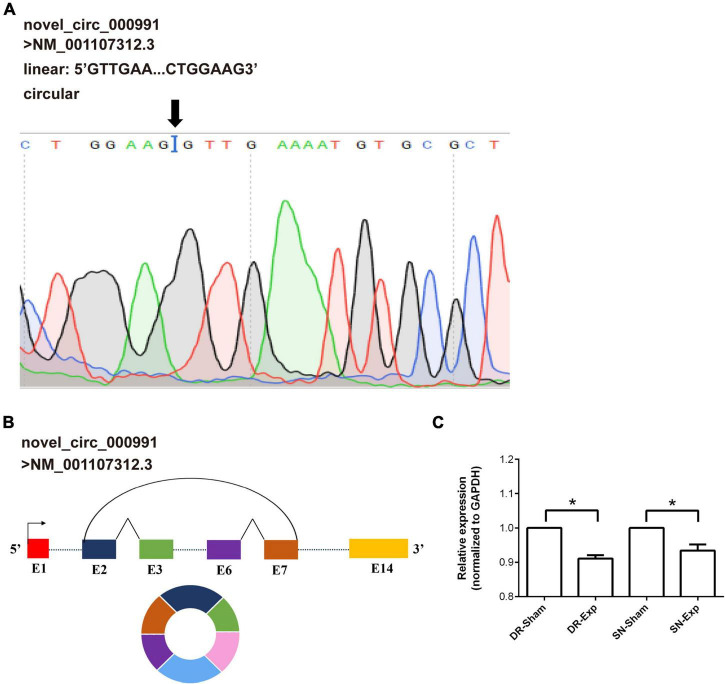
Validation of the characterization and expression of circular RNA novel_circ_000991. **(A)** Determination of novel_circ_000991 by Sanger sequencing. Black arrow indicates the site of back-splicing. **(B)** Schematic representation of the genomic locus of novel_circ_000991 in its host gene Mtmr7 (NM_001107312.3). **(C)** Relative expression levels of novel_circ_000991 after rat central and peripheral axon injuries. Data are summarized from 3 experiments and presented as mean with SEM. *P-value < 0.05.

Among these differentially expressed circular RNAs, circular RNA novel_circ_000991 exhibited relative high abundances in all groups and thus was further examined for its characterization and expression. Consistent with RNA sequencing outcome, Sanger sequencing recognized Mtmr7 (NM_001107312.3) as the host gene of novel_circ_000991 (Figure 4A). The cyclization site of novel_circ_000991 was demonstrated in a schematic diagram (Figure 4B). Moreover, the relative expression pattern of novel_circ_000991 was measured by RT-PCR. Sequencing data revealed a considerable reduction of the abundance of novel_circ_000991 after central axon branch injury and a decreasing trend (although not of statistical significance) after peripheral axon branch injury. RT-PCR results supported that central and peripheral axon injuries reduced novel_circ_000991 expression (Figure 4C).

### Functional analysis for host genes of differentially expressed circular RNAs following central axon injury

**FIGURE 5:**
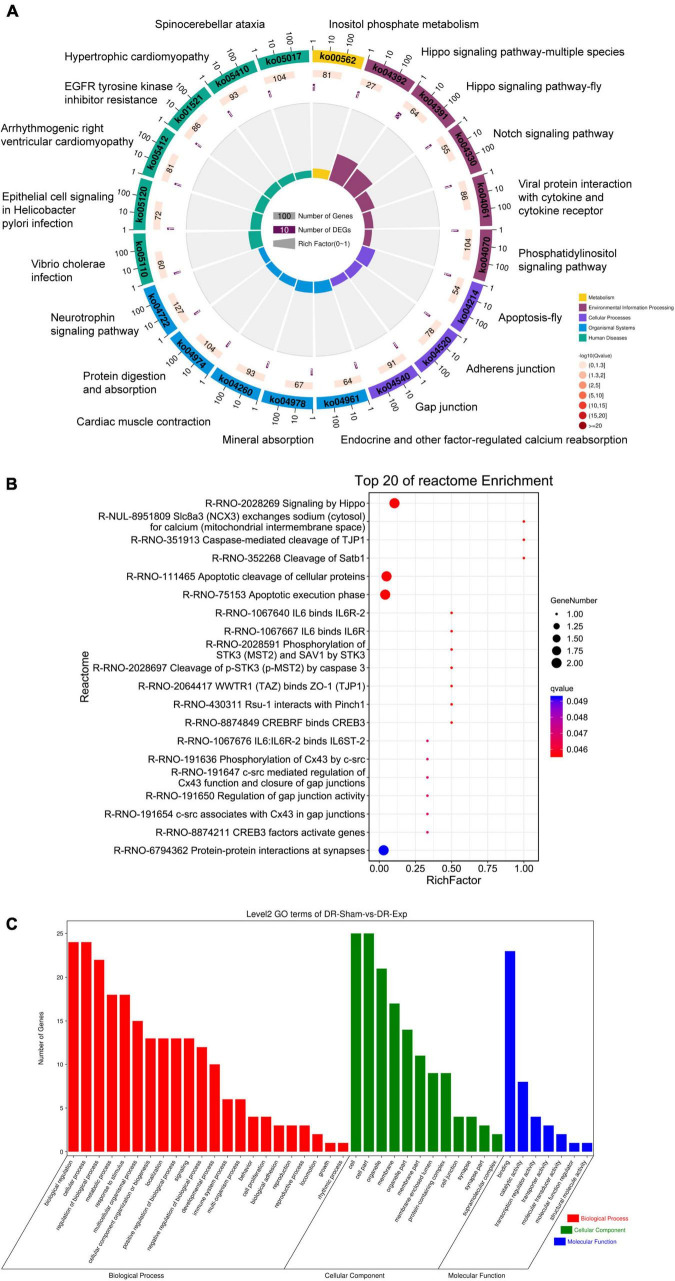
Annotation of host genes of differentially expressed circular RNAs after rat central axon injury. Enriched **(A)** KEGG pathways, **(B)** reactome pathways, and **(C)** GO terms.

To discover biological implications of differentially expressed circular RNAs in DRGs after central axon injury, host genes of these differentially expressed circular RNAs were annotated with KEGG database. Top enrich KEGG pathways were listed in Table 1 and displayed in a circular graph. A large number of development and regeneration-related pathways, such as Hippo signaling pathway (ko04391 and ko04392), Notch signaling pathway (ko04330), phosphatidylinositol signaling pathway (ko04070), and Neurotrophin signaling pathway (ko04722), were identified as significantly enriched KEGG pathways. Many cellular processes, including apoptosis (ko04214) and cellular junction-related pathways, i.e., adherens junction (ko04520) and gap junction (ko04540), were also found to be highly enriched (Figure 5A).

**TABLE 1:** Top 20 enriched kyoto encyclopedia of genes and genomes (KEGG) pathways for host genes of differentially expressed circular RNAs after rat central axon injury.

No	ID	Description
1	ko04391	Hippo signaling pathway -fly
2	ko04392	Hippo signaling pathway–multiple species
3	ko04214	Apoptosis–fly
4	ko04330	Notch signaling pathway
5	ko05110	Vibrio cholerae infection
6	ko04961	Endocrine and other factor-regulated calcium reabsorption
7	ko04978	Mineral absorption
8	ko05120	Epithelial cell signaling in Helicobacter pylori infection
9	ko04520	Adherens junction
10	ko00562	Inositol phosphate metabolism
11	ko05412	Arrhythmogenic right ventricular cardiomyopathy (ARVC)
12	ko01521	EGFR tyrosine kinase inhibitor resistance
13	ko04061	Viral protein interaction with cytokine and cytokine receptor
14	ko04540	Gap junction
15	ko04260	Cardiac muscle contraction
16	ko05410	Hypertrophic cardiomyopathy (HCM)
17	ko04070	Phosphatidylinositol signaling system
18	ko04974	Protein digestion and absorption
19	ko05017	Spinocerebellar ataxia
20	ko04722	Neurotrophin signaling pathway

Reactome analysis further demonstrated the considerable participation of Hippo signaling (Signaling by Hippo, R-RNO-2028269) and apoptosis (Apoptosis cleavage of cellular proteins, R-RNO-111465; Apoptotic execution phase, R-RNO-75153; and Cleavage of p-STK3 (p-MST2) by caspase 3, R-RNO-2028697) in DRGs after central axon injury. The enrichment of reactome pathways WWTR1 (TAZ) binds to ZO-1 (TJP1) (R-RNO-2064417), phosphorylation of Cx43 by c-src (R-RNO-191636), c-src mediated regulation of Cx43 function and closure of gap junctions (R-RNO-191647), and regulation of gap junction activity (R-RNO-191654) implied changes of cellular junction. The identification of significantly involved reactome pathway Caspase-mediated cleavage of TJP1 (R-RNO-351913) further indicated the association of apoptosis and cellular junction regulation. Moreover, the recognition of reactome pathways IL6 binds IL6R-2 (R-RNO-1067640), IL6 binds IL6R (R-RNO-1067667), and IL6:IL6R-2 binds IL6ST-2 (R-RNO-1067676) illuminated the involvement of immune responses in central axon injury-mediated molecular features (Figure 5B).

Gene ontology term enrichment revealed the involvement of GO biological processes biological regulation, cellular process, regulation of biological process, GO cellular components cell, cell part, and GO molecular function binding (Figure 5C).

### Functional analysis for host genes of differentially expressed circular RNAs following peripheral axon injury

**FIGURE 6:**
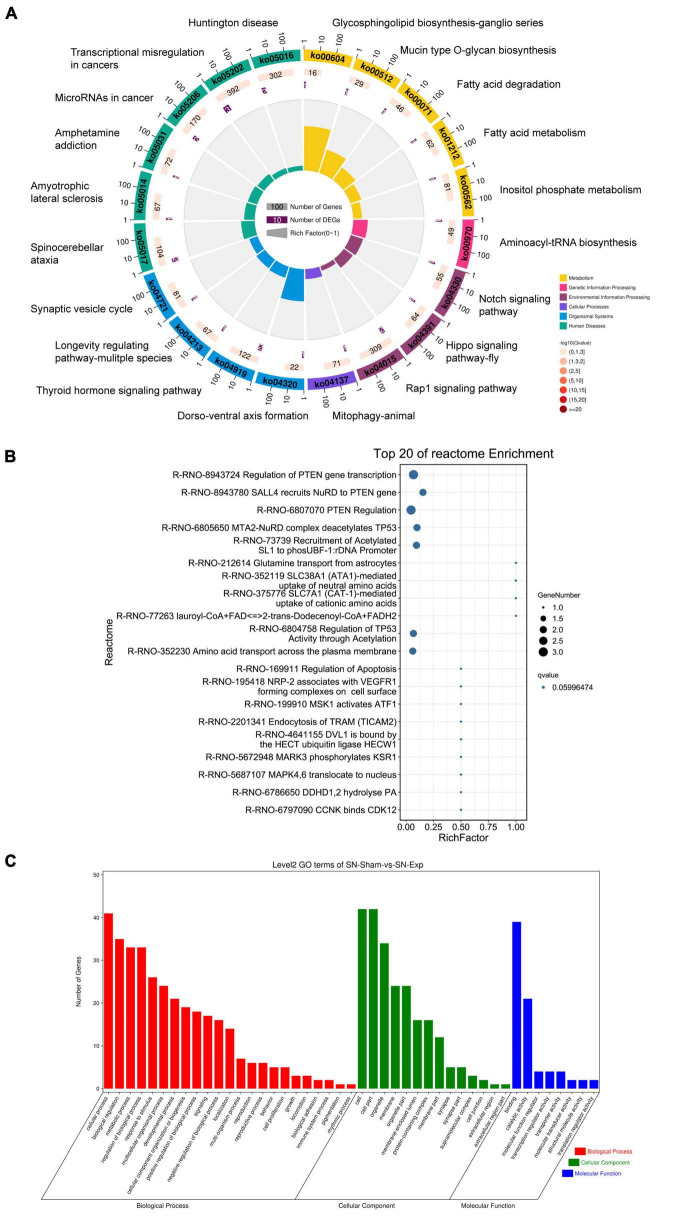
Annotation of host genes of differentially expressed circular RNAs after rat peripheral axon injury. Enriched **(A)** KEGG pathways, **(B)** reactome pathways, and **(C)** GO terms.

Host genes of differentially expressed circular RNAs in DRGs after peripheral axon injury were subjected to functional enrichment as well. Similar as changes after central axon injury, Hippo signaling pathway and Notch signaling pathway were found to be enriched after peripheral axon injury. A metabolism-related pathway, Inositol phosphate metabolism (ko00562), was also discovered as a commonly involved KEGG pathway in host genes of differentially expressed circular RNAs after both central and peripheral axon injuries. Besides Inositol phosphate metabolism, many other metabolism-related pathways, i.e., Glycosphingolipid biosynthesis-ganglio series (ko00604), Mucin type O-glycan biosynthesis (ko00512), Fatty acid degradation (ko00071), and Fatty acid metabolism (ko01212) were also significantly changed in DRGs after peripheral axon injury (Table 2 and Figure 6A).

**TABLE 2:** Top 20 enriched kyoto encyclopedia of genes and genomes (KEGG) pathways for host genes of differentially expressed circular RNAs after rat peripheral axon injury.

No	ID	Description
1	ko05017	Spinocerebellar ataxia
2	ko04919	Thyroid hormone signaling pathway
3	ko00604	Glycosphingolipid biosynthesis–ganglio series
4	ko04320	Dorso-ventral axis formation
5	ko05206	MicroRNAs in cancer
6	ko00512	Mucin type O-glycan biosynthesis
7	ko05202	Transcriptional misregulation in cancers
8	ko00071	Fatty acid degradation
9	ko00970	Aminoacyl-tRNA biosynthesis
10	ko04330	Notch signaling pathway
11	ko01212	Fatty acid metabolism
12	ko04391	Hippo signaling pathway -fly
13	ko04213	Longevity regulating pathway–multiple species
14	ko05014	Amyotrophic lateral sclerosis (ALS)
15	ko04137	Mitophagy–animal
16	ko05031	Amphetamine addiction
17	ko05016	Huntington disease
18	ko04015	Rap1 signaling pathway
19	ko00562	Inositol phosphate metabolism
20	ko04721	Synaptic vesicle cycle

Reactome enrichment analysis showed the essential involvement of PTEN in peripheral axon injury-induced changes in DRGs as many significant reactome pathways were related to PTEN, including Regulation of PTEN gene transcription (R-RNO-8943724), SALL4 recruits NuRD to PTEN gene (R-RNO-8943780), and PTEN Regulation (R-RNO-6807070). Furthermore, TP53-related reactome pathways MTA2-NuRD complex deacetylates TP53 (R-RNO-6805650) and Regulation of TP53 activity through acetylation (R-RNO-6804758) as well as ATF-associated pathway MSK1 activates ATF1 (R-RNO-6804758) were also identified as top enriched reactome pathways (Figure 6B).

Gene ontology analysis revealed cellular process as the most significantly enriched GO biological process, cell and cell part as the most significantly enriched GO cellular components, and binding as the most significantly enriched GO molecular function (Figure 6C).

**FIGURE 7:**
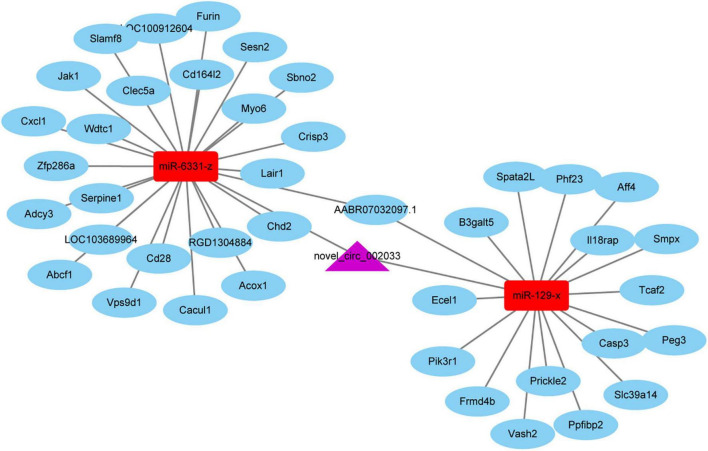
The competing endogenous RNA (ceRNA) network of novel_circ_002033. Purple indicates circular RNA novel_circ_002033, red indicates microRNAs miR-6331-z and miR-129-x, and blue indicates target genes.

Given the essential participation of PTEN-related pathways in peripheral axon injury-induced changes, circular RNAs sourced from genes involved in PTEN pathways are worthy of note. Novel_circ_002033, a circular RNA cyclizated from Gatad2b, was found to be up-regulated after peripheral axon branch injury versus sham surgery but not significantly altered after central axon branch injury. Therefore, besides the function of the host gene of novel_circ_002033, the regulatory roles of novel_circ_002033 was also explored by generating the ceRNA network of novel_circ_002033. Regulatory network showed that novel_circ_002033 might function as a microRNA sponge and regulate the target genes of miR-6331-z (i.e., Abcf1, Serpine1, Cxcl1, Zfp286a, Adcy3, Wdtc1, Slamf8, Acox1, Cd16412, Cacul1, Cd28, LOC100912604, Jak1, Furin, Myo6, Chd2, Crips3, Sbno2, RGD1304884, AABR07032097.1, Clec5a, Lair1, Vps9d1, Sesn2, and LOC103689964) and miR-129-x (i.e., Vash2, Aff4, Smpx, Frmd4b, Slc39a14, Casp3, Prickle2, Peg3, Spata2L, Phf23, Pik3r1, Ecel1, Ppfibp2, AABR07032097.1, Tcaf2, B3galt5, and Il18rap) (Figure 7).

## Discussion

Dorsal root ganglion neurons are extensively used to investigate axon regeneration as the central and peripheral axon branches of DRG neurons have different axon regrowth abilities (Chen et al., 2016). Identifying different injury responses in DRGs after central and peripheral axon branches help to illuminate the molecular basis of successful axon regeneration and provides prospects for the treatment of central nerve injury.

Previously, our laboratory has investigated changes in DRGs at a series of time points, i.e., 0 h, 3 h, 9 h, 24 h, 4, and 7 days after rat sciatic nerve injury and found that many genes exhibited altered expression levels at 24 h post peripheral axon injury (Gong et al., 2016). To examine differences between peripheral and central axon injuries, herein, we performed rat central and peripheral axon injuries, collected DRGs at 24 h after nerve injury, and observed injury-induced changes in DRGs from the aspect of circular RNAs. The database of rat circular RNA is not as comprehensive as human circular RNAs, many identified circular RNAs in rat DRGs are thus labeled as novel circular RNAs. The genomic origins and localizations of these circular RNAs demonstrate that circular RNAs are chiefly derived from exonic regions and largely transcribed from rat chromosome 1, which is consistent with observations of circular RNAs in DRGs after sciatic nerve injury (Mao et al., 2019). Similar as the findings of the amounts of changed coding genes and accessible genes (Palmisano et al., 2019), the numbers of differentially expressed circular RNAs in DRGs are much larger after peripheral axon injury as compared with that in DRGs after central axon injury, indicating that sciatic nerve injury-induced changes may be more remarkable. Among these differentially expressed genes, only one circular RNA, that is novel_circ_000986, a circular RNA derived from linear Tshz2, is found to be up-regulated after both central and peripheral axon injuries, and only one circular RNA, that is novel_circ_000151, a circular RNA derived from linear Tesk2, is consistently down-regulated. The low-degree of genetic overlapping indicates that nerve injuries at central and peripheral axon branches induce diverse injury responses.

Functional analyses of host genes of circular RNAs provide important biological implications as circular RNAs regulate their host genes (Zhou et al., 2022). Hippo signaling pathway, a regeneration-promoting signaling pathway that orchestrates neural crest cell development and glial cell expansion (Serinagaoglu et al., 2015; Jeanette et al., 2021; Zhao et al., 2021) and Notch signaling pathway, a regeneration-inhibiting signaling pathway that prevents growth cone formation (El Bejjani and Hammarlund, 2012), are commonly involved after central and peripheral axon injuries. Besides the recognization of concurrent changes, the identification of unique signaling pathways may also be very meaningful as it reflects different responses to central and peripheral injuries. KEGG and reactome pathway annotation show the enrichment of apoptosis and cellular junction after central axon injury instead of peripheral axon injury. Significant involvement of apoptosis after central axon injury is in agreement with experimental observations that spinal cord injury elicit severe neuron loss while sciatic nerve injury only lead to the death of 10–30% sensory neurons (Navarro et al., 2007; Park et al., 2008). Therefore, improving neuronal survival may be effective for the treatment of central nerve injury. Changes of blood-nerve-barrier have been recently detected in post-injury sensory neuron microenvironment by single cell sequencing (Avraham et al., 2021). Here, the significant involvements of adherens junction, gap junction, and more importantly, the mediation of tight junction protein 1 (TJP1, ZO-1) by caspase in central axon injury-induced alterations indicate that elevated apoptosis may contribute to cellular junction disruption and the remodeling of neuron microenvironment.

The most distinctive feature of host genes of differentially expressed circular RNAs after peripheral axon injury is the enrichment of PTEN-related pathways. PTEN/mTOR pathway has been long recognized as an essential regulator of nerve regeneration (Park et al., 2008; Abe et al., 2010; Christie et al., 2010; Liu et al., 2010). Endosomal NADPH oxidase 2, a DRG regenerative outgrowth contributing factor identified by comparing coding genes in DRGs after central and peripheral axon injuries, executes a promoting role in nerve regeneration *via* PTEN oxidation and inactivation (Hervera et al., 2018). Therefore, both analyses of coding genes and non-coding circular RNAs reveal the significance of PTEN signaling and indicate that inactivated PTEN may be a prerequisite for success axon regeneration.

Circular RNAs often function as ceRNA sponges, competitively bind to microRNAs, and increase the abundances of target genes of these microRNAs (Hansen et al., 2013; Li et al., 2018). By analyzing the targeting relationship and expression correlations of circular RNAs, microRNAs, and mRNAs, ceRNA networks were constructed and many circular RNAs that might be involved in ceRNA networks were screened, such as novel_circ_000013, novel_circ_000020, and novel_circ_000047. A full list of correlated circular RNAs, microRNAs, and mRNAs was provided in Supplementary Table 3. Among involved circular RNAs, novel_circ_002033 is cyclized from linear Gatad2b, a gene involved in PTEN signaling. Therefore, we investigated a novel_circ_002033-centered ceRNA network in detail. Notably, some downstream target genes in the ceRNA network have already been reported to be complicated in the nerve regeneration process. For instance, Acox1, a downstream target molecule of miR-6331-z, is required for axon regeneration and its mutation induces axonal loss and axon degeneration (Chung et al., 2020; Griffin and Ackerman, 2020; Shimizu et al., 2022). Ecel1, a target of miR-129-x, displays robust transcriptional responses to nerve injury and is considered as a potential therapeutic target for nerve regeneration (Kiryu-Seo et al., 2019). Therefore, other molecules in the constructed ceRNA network with currently unknown functions may also play important roles in nerve repair and regeneration and are worth further investigating.

Taken together, in the present study, we investigated novel circular RNA expression profiles of DRGs following nerve injury and discovered unique responses of DRGs to central and peripheral projecting axon injuries. These identified genetic signatures in DRGs may benefit the understanding of molecular changes underlying failed central axon injury and success peripheral axon injury and contribute to the development of therapeutic strategies for central nerve regeneration.

## Data availability statement

The datasets presented in this study can be found in online repositories. The names of the repository/repositories and accession number(s) can be found in the article/Supplementary material.

## Ethics statement

The animal study was reviewed and approved by Nantong University Institutional Animal Care and Ethical approved by the Administration Committee of Experimental Animals, Nantong University, Jiangsu Province, China.

## Author contributions

H-JC, LH, M-RZ, and TZ performed the experiment. L-CX designed the experiment. All authors wrote the manuscript.

## Supplementary material

Supplementary Table 1 List of differentially expressed circular RNAs after rat central axon injury.

Supplementary Table 2 List of differentially expressed circular RNAs after rat peripheral axon injury.

Supplementary Table 3 List of predicted correlations between circular RNAs, microRNAs, and mRNAs.
